# HHV‐8/KSHV in Solid Organ Transplantation: Current Gaps of Knowledge and Future Directions

**DOI:** 10.1111/tid.70179

**Published:** 2026-01-30

**Authors:** Alessandra Mularoni, Andrea Cona, Malgorzata Mikulska, Francesca Pecoraro, Carlotta Piazza, Elda De Vita, Giada Pietrosi, Matteo Bulati, Tiziana Lazzarotto, Mario Luppi

**Affiliations:** ^1^ IRCCS ISMETT Palermo Italy; ^2^ UPMC Italy Palermo Italy; ^3^ Division of Infectious Diseases Department of Health Sciences University of Genoa Genoa Italy; ^4^ IRCCS Ospedale Policlinico San Martino Genoa Italy; ^5^ Department of Medical and Surgical Sciences Alma Mater Studiorum University of Bologna Bologna Italy; ^6^ Microbiology Unit IRCCS Azienda Ospedaliero‐Universitaria di Bologna Bologna Italy; ^7^ Section of Hematology Department of Medical and Surgical Sciences University of Modena and Reggio Emilia AOU Modena Modena Italy

**Keywords:** Diffuse large B‐cell lymphoma, human herpes virus 8/Kaposi's sarcoma‐associated herpesvirus, Kaposi sarcoma, Kaposi sarcoma inflammatory cytokine syndrome, multicentric Castleman disease, primary effusion lymphoma, solid organ transplantation

## Abstract

The incidence of HHV‐8/KSHV–associated diseases (KADs) among solid organ transplant (SOT) recipients has shown a relative increase, likely reflecting the growing population of long‐term SOT survivors and heightened recognition and reporting due to greater clinician awareness. The real impact of HHV‐8/KSHV in the SOT setting remains difficult to determine due to regional variations in seroprevalence and non‐universal screening practices. Neoplastic KAD reported in SOT includes Kaposi sarcoma (KS), multicentric Castleman disease (MCD), primary effusion lymphoma (PEL), and other rare lymphomas. Increasing attention has focused on Kaposi's Sarcoma‐associated Herpesvirus Inflammatory Cytokine Syndrome (KICS), a non‐malignant syndrome characterized by uncontrolled inflammation, fever, pancytopenia and elevated HHV‐8/KSHV DNAemia, resembling viral sepsis that can progress to shock and multi‐organ failure. A clinical protocol including testing donors and recipients, monitoring for DNAemia in recipients at risk, switching CNI to mTOR inhibitors, treatment with antivirals, and rituximab for KICS may mitigate the impact of HHV‐8/KSHV infection in SOT recipients. However, standardized serological testing is not available and the role of monitoring, preemptive management and treatment of HHV‐8/KSHV DNAemia should be studied in larger prospective studies. Severe donor‐derived KICS and KS, often presenting without skin involvement, underscore the need for reliable serologic tests for identification of at‐risk recipients (especially D+/R‐), heightened clinical awareness to ensure timely diagnosis and prompt treatment. This review provides an updated overview of KADs with a particular focus on KICS in SOT, highlights knowledge gaps for future research, and summarizes recent advances in the screening and management of HHV‐8/KSHV infection following transplantation.

## Graphical abstract

Risk mitigation strategies, including donors/recipients screening, DNAemia monitoring in recipients at risk, CNI‐to‐mTOR inhibitors switch, antivirals, and rituximab for KICS, may mitigate the impact of HHV‐8/KSHV infection in SOT. This review provides an update on KICS, identifies research gaps, and summarizes advances in screening and management.

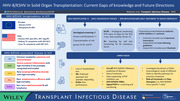

**Abbreviations**
CDC: Center for Disease Control
CNI: Calcineurin inhibitor
DLBCL‐NOS: Diffuse large B cell lymphoma not otherwise specified
CRP: C‐reactive protein
HHV‐8/KSHV: Human herpes virus 8/Kaposi's sarcoma‐associated herpesvirus
D: donor
EBV: Epstein‐Barr virus
HSCT: Hematopoietic stem‐cell transplant
KAD: HHV‐8/KSHV‐associated disease
IFN: interferon
KICS: Kaposi's Sarcoma‐associated Herpesvirus Inflammatory Cytokine Syndrome
KS: Kaposi sarcoma
LANA: Latency‐associated nuclear antigen
MCD: Multicentric Castleman Disease
MOF: Multiorgan failure
MSM: Men who have sex with men
mTOR: Mammalian target of rapamycin
PEL: Primary effusion lymphoma
PET/CT: Positron emission tomography/computed tomography
PT: Post‐transplant
PT‐KS: Post‐transplant Kaposi sarcoma
R: Recipient
SOT: Solid organ transplant
TNF: Tumor necrosis factor

## 1 Introduction

Initially identified by Yuan Chang and Patrick Moore in 1994, human herpesvirus 8 (HHV‐8), also known as Kaposi's sarcoma‐associated herpesvirus (KSHV), is a gamma‐herpesvirus with a lifecycle characterized by lytic and latent phases [1]. This virus can cause a spectrum of neoplastic and inflammatory disorders, predominantly affecting immunocompromised individuals [2].

The seroprevalence of HHV‐8/KSHV differs by geographic regions and risk groups (Table 1); in particular, it ranges between 5% in North Europe, 3%–7% in the U.S., 20%–30% in the Mediterranean, and more than 50% in Sub‐Saharan Africa [2]. However, even in low endemic areas, like the U.S., seroprevalence ranges between 40% and 60% in people living with HIV and men who have sex with men (MSM). While the incidence of HHV‐8/KSHV‐associated diseases (KADs) in individuals living with HIV has been steadily declining due to the widespread adoption of effective antiretroviral therapy and improved immune reconstitution, the incidence of KADs in solid organ transplant (SOT) recipients has shown a relative increase due to the growing population of SOT recipients with prolonged life expectancy. On September 30th 2025, the Center for Disease Control (CDC) released an important update regarding ongoing investigations into increased reports of suspected organ donor‐derived HHV‐8/KSHV in SOT recipient, resulting in Kaposi sarcoma (KS), Kaposi's sarcoma‐associated herpesvirus inflammatory cytokine syndrome (KICS), or other KADs [3]. This trend underscores the critical need for intensified surveillance and high levels of vigilance, as well as the importance of reporting suspected donor‐derived infections.

**TABLE 1:** Seroprevalence of HHV‐8/KSHV infection in SOT.

Author, Journal, Year, Ref	Country	Type of Transplant	Seroprevalence (%)
Europe			
Marcelin AG. et al. [4], *Liver Transplant*, 2004	France	Liver	Donors 3.3%, Recipients 2.5%
García‐Astudillo LA. et al. [5], *Transplant Immunology*, 2006	Spain	Liver and Kidney	Liver 3.4%, Kidney 0.6%
Frances C. et al. [6], *American Journal of Transplantation*, 2009	France	Kidney	Donors 1.1%, Recipients 3.2%
Pietrosi G. et al. [7], *American Journal of Transplantation*, 2011	Italy	Liver	Donors 4.4%, Recipients 10.2%
Lebbe C. et al. [8], *American Journal of Transplantation*, 2013	Italy	Liver, Kidney, Heart	Donors 12%, Recipients NA
Chiereghin A. et al. [9], *Transplantation*, 2017	Italy	Kidney, Liver, Heart	Donors: 4%, Recipients: 18%
Mularoni A. et al. [10], *American Journal of Transplantation*, 2025	Italy	Kidney, Liver, Heart, Lung	Donors 3.3 %, Recipients 8.4% Liver 10.2% Kidney 6.3% Heart 6.6% Lung 6.9%
Roo‐Brand G et al. [11]*, Journal of Medical Virology*, 2025	Netherland	Kidney, Liver, Heart, Lung, Pancreas, Small intestine	Donors: 2.8%, Recipients: 10.3% Liver 8.5% Kidney 10.7% Heart 12.5% Lung 10% Pancreas 33.3% Small Intestine 0%
US			
Jenkins FJ. et al. [12], *Journal of Infectious Diseases*, 2002	US	Multi‐organ	Liver 21.7% Kidney 15.8% Heart 23% Lung 20% Multi‐organ 16.7%
Durand C. et al. [13], *American Journal of Transplantation*, 2022	US	Liver	HIV+ Liver Recipients: 21% HIV+ Donors: 25%
Nambiar P. et al. [14], *Clinical Infectious Diseases*, 2025	US	Kidney	HIV+ kidney Recipients: 40.6% HIV+ Donors: 25.2%
Global			
Bonazzetti C. et al. [15], *Clinical Microbiology and Infection*, 2025	Systematic review	Multi‐organ	Donors 3.6%, Recipients 5.8%

In the context of SOT, the timing, type of presentation, and severity of KADs are influenced by several factors. These include the donors’ and recipients’ pre‐transplant serological status, since primary infections are more often associated with inflammatory manifestations and a more aggressive visceral form of KS, as well as the degree of immunosuppression and the type of organ transplanted. Notably, lung and liver transplants tend to be linked to more severe disease forms and poorer outcomes [7, 10, 16, 17].

Early recognition of these clinical manifestations is critical, as reducing and/or modifying immunosuppression and initiating appropriate therapies without delay (e.g. rituximab, antiviral agents, and chemotherapy) can be lifesaving. The management of HHV‐8/KSHV infection in SOT recipients remains particularly challenging: First, diagnosis is often difficult, as the infection can present with non‐specific or atypical symptoms (e.g. blood cultures negative or viral sepsis) and may evolve rapidly with fatal outcome, if not adequately and promptly treated; second, the available serological assays are not standardized and are operator‐dependent, leading to variability in sensitivity and specificity across laboratories (Table 2). Furthermore, current management strategies are largely informed by single‐center case series or retrospective analyses, with a lack of robust, multicenter prospective studies to guide evidence‐based practice.

**TABLE 2:** Sensitivity and specificity of available HHV‐8/KSHV serological assays used in literature in SOT.

Author, Country, year	Sample size	Advanced Biotechnologies Inc (ABI)—IFA *(HHV‐8 infected cells)*	Biotrin IFA *(lytic antigens)*	SCIMEDX IFA *(latent and lytic antigens)*	Advanced Biotechnologies Inc (ABI)—ELISA *(purified HHV‐8 virions)*
**Pietrosi G. et al**. [7]**, Italy, 2011**	**179 Liver donors and recipients** **D+/R− = 8** **R+ = 19** **D−/R− = 152**	ND	ND	**Latent antigens**: *Sensitivity: 80%–90%* *Specificity: 97%*	**Lytic plus latent antigens**: *Sensitivity*: 80%–90% *Specificity*: 89%
**Lebbe C. et al**. [8]**, France, 2013**	**2354 donors (seroprevalence 12%)** **D+/R− = 454**	ND	**Latent antigens (home‐made test)**: *Sensitivity: 61%–72%* *Specificity: 99%–100%* **Lytic antigens**: *Sensitivity: 100%* *Specificity: 94%*	ND	ND
**Chiereghin A. et al**. [9]**, Italy, 2017**	**Pre‐Tx screening in donors and recipients with 6 serological assays (4 IFA and 2 ELISA lytic and latent antigen based)** ***R* = 517** ***D* = 249**	***Donors HHV‐8/KSHV Ab positive***: 10/249 (4%) ***Recipients HHV‐8/KSHV Ab positive***: 93/517 (18%) **Lytic plus latent antigens**: *Sensitivity*: 98% *Specificity*: 98.3% *ABI IFA and Biotrin IFA tests showed almost perfect agreement to the reference standard (0.943 and 0.931 Cohen kappa, respectively)*. *Scimedx lytic IFA test showed a substantial agreement (0.831)*	***Donors HHV‐8/KSHV*** ***Ab positive***: 10/249 (4%) ***Recipients HHV‐8/KSHV Ab positive***: 82/517 (16%)	***Donors HHV‐8/KSHV Ab positive for lytic antigens***: 10/249 (4%) ***Donors HHV‐8/KSHV Ab positive for latent antigens***: 5/249 (2%) ***Recipients HHV‐8/KSHV Ab positive for lytic antigens***: 66/517 (12.8%) ***Recipients HHV‐8/KSHV Ab positive for latent antigens***: 34/517 (6.6%) **Lytic antigens**: *Sensitivity*: 77.6% *Specificity*: 99.2% **Latent antigens**: *Sensitivity: 37.8%* *Specificity: 99.8%*	***Donors HHV‐8/KSHV Ab positive***: 4/249 (1.6%) ***Recipients HHV‐8/KSHV Ab positive***: 54/517 (10.4%) **Lytic plus latent antigens**: *Sensitivity: 51.4%* *Specificity: 99.4%* *Scimedx latent IFA and ABI ELISA tests had a lesser agreement (0.509 and 0.639 Cohen kappa, respectively)*.
**Leducq V. et al**. [4]**, France, 2024**	**Comparison of IFA and ELISA** **Positive control groups** (*n* = 49) **Negative control groups** (*n* = 14) **Equivocal IFA results group** (*n* = 14)	ND	**Latent antigens (home‐made test)**: *Sensitivity*: 79% *Specificity*: 100%	ND	**Lytic plus latent antigens**: *Sensitivity: 94% Specificity: 100%*

This review, performed using a search strategy in MEDLINE with predefined keywords until September 2025, aims to provide an updated overview of current knowledge regarding HHV‐8/KSHV infection in the SOT setting, with particular attention to KICS and risk mitigation strategies, underlying the opportunities and emphasizing limitations related to serological screening of donors and recipients.

## 2 HHV‐8/KSHV Characteristics, Life Cycle, and Prevalence of Infection

HHV‐8/KSHV is a double‐stranded DNA virus infecting B cells, endothelial cells, macrophages, and monocytes. While saliva is the primary route of transmission, the virus can also be spread through sexual contact, vertical transmission, and intravenous drug use. In addition, iatrogenic transmission has been documented, particularly through blood transfusion and SOT [2].

Upon entry into the host, HHV‐8/KSHV virions bind to host cell surface receptors through viral glycoproteins, such as gpK8.1‐S, facilitating subsequent endocytosis into the cell.

**FIGURE 1:**
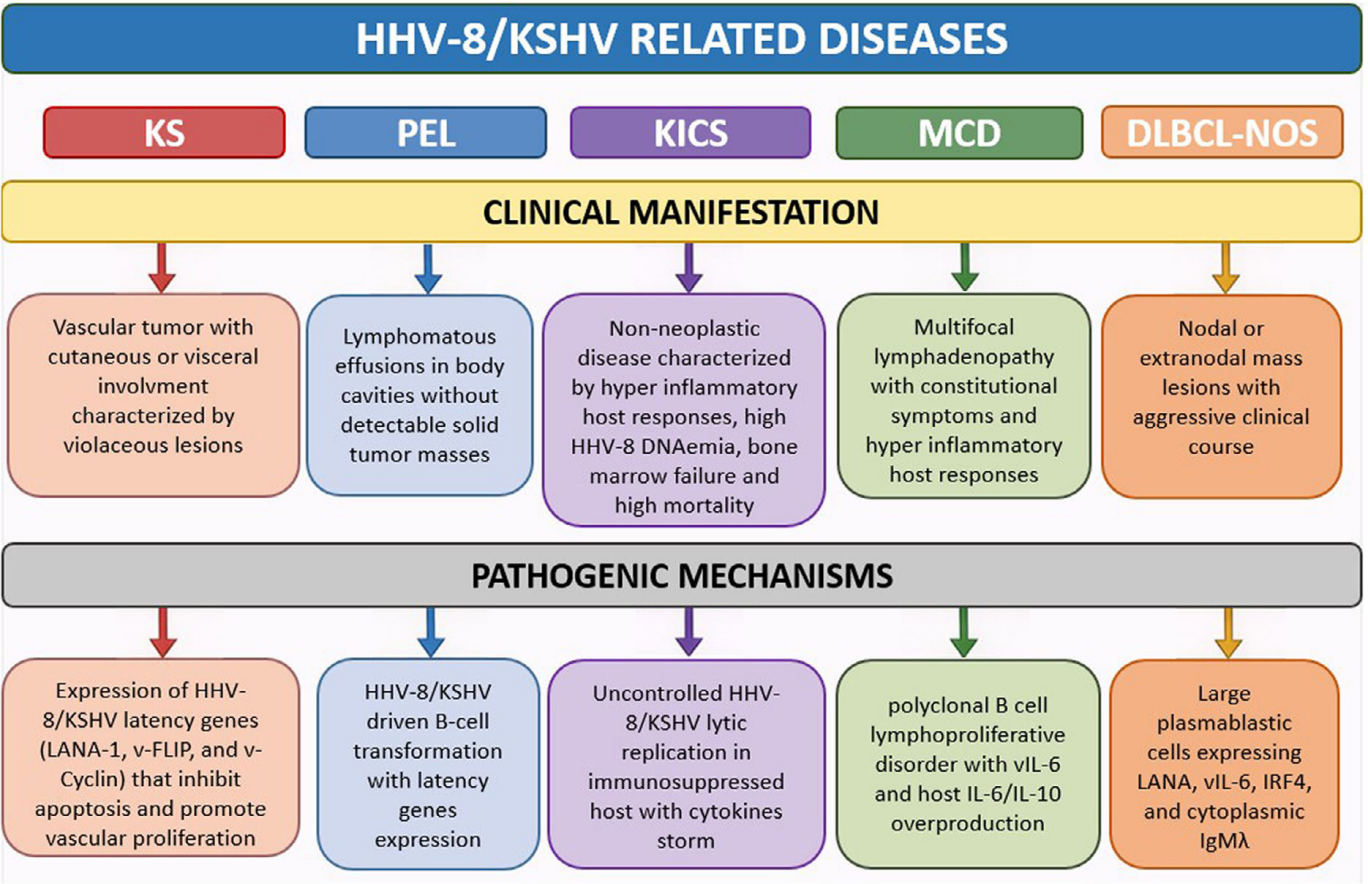
Clinical spectrum of HHV‐8/KSHV‐related diseases in the SOT setting.

The life cycle of HHV‐8/KSHV is similar to that of other herpesviruses. During latency, HHV‐8/KSHV expresses a restricted set of genes promoting viral persistence and proliferation by inhibiting apoptosis and promoting angiogenesis. These processes are key in the pathogenesis of HHV‐8/KSHV associated neoplastic diseases and lymphoproliferative disorders, such as KS, Primary Effusion Lymphoma (PEL), HHV‐8/KSHV‐associated diffuse large B‐cell lymphoma, not otherwise specified (DLBCL‐NOS), and multicentric Castleman disease (MCD) of plasma cell type [18, 19, 20] (Figure 1).

Conversely, the activation of the virus's lytic cycle begins with the immediate‐early transactivator protein RTA expression, triggering viral DNA replication, and virion assembly and release. This phase is characterized by the production of viral interleukins and chemokines, causing systemic inflammation and angiogenesis [18].

In the setting of SOT, data on seroprevalence are sparse and derived from single‐center studies [8, 9, 10, 11], since current guidelines do not recommend universal screening mainly due to the lack of standardized serologic assays and uncertainty about the best preventive strategies [21]. An international survey on the screening and management of HHV‐8/KSHV infection in SOT revealed that only 33% of centers conducted pre‐transplant serological testing [22]. The rates varied, with 37.5% of centers in high HHV‐8/KSHV prevalence regions performing the test, compared to 0% in low‐prevalence areas. Post‐transplant (PT) HHV‐8/KSHV DNA monitoring was reported in 21 centers (41%). Notably, centers that carried out screening and monitoring were more likely to have diagnosed a nonmalignant KAD within the previous five years [22]. Recent European studies report that approximately 3%–4% of donors and 8%–18% of recipients are HHV‐8/KSHV seropositive [8, 9, 10, 11]. In our recent study, liver transplant patients exhibited the highest seroprevalence rates (10%), compared to lower rates observed in lung (7%), kidney (6%), and heart (6%) recipients, respectively. The two most recent studies conducted by Durand et al. and Nambiar et al., within the HOPE cohort in the United States, observed a HHV‐8/KSHV seroprevalence of 21% and 41% in HIV‐infected liver and kidney recipients [13, 14], respectively, and 25% among donors with HIV; notably, seroprevalence of donors without HIV was 8% [14].

While usually asymptomatic in immunocompetent hosts, HHV‐8/KSHV primary infection or reactivation in immunocompromised patients can lead to a spectrum of diseases, ranging from malignancies to severe inflammatory syndromes [23, 24, 25].

## 3 HHV‐8/KHSV‐Associated Neoplastic Diseases

### 3.1 Kaposi Sarcoma

KS is the most common HHV‐8/KSHV‐associated malignancy and usually manifests with painless, single, or multiple violaceous lesions, raised or flat, that can appear in any part of the skin including surgical scars (“Koebner phenomenon”)[2, 26, 27]. SOT recipients are at 400‐fold increased risk as compared to the general population [28]. In post‐transplant Kaposi sarcoma (PT‐KS), involvement of lymph nodes is present in 20%–40% of cases while visceral involvement in 20%–50% of cases, usually with gastrointestinal, respiratory tract, or graft involvement [29]. Atypical PT‐KS presentations have been reported, including tonsillar KS successfully treated with tonsillectomy and sirolimus, and visceral forms involving the bladder wall and pelvic lymph nodes [30, 31]. Diagnosis relies on histological confirmation, characterized by HHV‐8/KSHV LANA‐positive spindle cells. Imaging, endoscopy, and positron emission tomography/computed tomography (PET/CT) are useful for staging of visceral involvement. Visceral KS without skin lesions is not uncommonly reported in SOT recipients and presents diagnostic challenges, particularly when symptoms mimic other PT complications.

The cornerstone of treatment involves reduction or modification of immunosuppression, with a switch from calcineurin inhibitors (CNI), such as cyclosporine or tacrolimus, to mammalian target of rapamycin inhibitors (mTORi), like sirolimus or everolimus. In more extensive or visceral KS, chemotherapy is employed with Pegylated liposomal doxorubicin or, as second line therapy, paclitaxel [27]. In conclusion, PT‐KS frequently presents with atypical, aggressive, and visceral manifestations. Donor‐derived infections are associated with earlier onset and more aggressive, often fatal, visceral KS, whereas seropositive recipients can develop both cutaneous and visceral lesions with later onset.

These findings underscore the critical importance of early recognition, implementation of HHV‐8/KSHV screening protocols, and individualized management strategies tailored to patient risk profiles [26, 30, 31, 32, 33]. In fact, cutaneous and promptly diagnosed KS often resolve with switch to mTORi alone [30, 31, 33], whereas disseminated KS typically requires chemotherapy and is associated with a higher risk of fatal outcomes [16, 26].

### 3.2 PEL and Diffuse Large B‐cell Lymphoma Not Otherwise Specified

PEL is a rare but highly aggressive form of non‐Hodgkin lymphoma, usually affecting immunocompromised hosts, typically characterized by lymphomatous effusions accumulating in body cavities such as the pleural, peritoneal, or pericardial spaces, without detectable solid tumor masses. Atypical presentations such as refractory ascites and solid tumor variants have been documented, highlighting diagnostic challenges [2].

In a recent systematic review, 13 PT‐PEL cases were described: 6 in kidney, 3 in heart, 2 in liver, 1 in bowel transplant recipients, and 1 hematopoietic stem cell transplant (HSCT) recipient. In addition to PEL, which developed in median 8 years PT, KS was also diagnosed in 4 patients. Despite treatment, all cases were fatal within few months [34].

In addition to PEL, HHV‐8/KSHV has also been implicated in the development of DLBCL‐NOS. This rare entity typically manifests with solid tumor masses, and often involves lymph nodes or extranodal sites. Like PEL, DLBCL‐NOS tends to have an aggressive clinical course, with poor prognosis in most cases, particularly in immunocompromised patients [34]. Only 2 cases of HHV‐8/KSHV‐DLBCL‐NOS have been reported in SOT recipients. One patient with concomitant KS achieved remission after treatment, while in the other case was diagnosed with DLBCL two months after KS and succumbed despite therapy [35, 36].

### 3.3 Multicentric Castleman Disease

MCD is a polyclonal B cell lymphoproliferative disorder characterized by multifocal lymphadenopathy and systemic hyper‐inflammatory host responses, driven by excessive cytokine production. This hyper‐inflammatory state is characterized by persistent fever, night sweats, weight loss, and profound fatigue. Over time, uncontrolled immune activation may result in bone marrow suppression and multi‐organ failure (MOF). MCD can progress rapidly and require targeted biological therapies to control the disease and prevent fatal outcomes.

A recent review reported 10 cases of PT‐MCD including 6 kidney, 3 liver, and 1 heart transplant patients. Disease occurred approximately 4 years PT. Four cases had concurrent KS, and 1 was associated with an HHV‐8/KSHV–related lymphoproliferative disorder. Clinical presentations ranged from asymptomatic to widespread lymphadenopathy, splenomegaly, and systemic symptoms. Most patients exhibiting aggressive systemic disease and receiving only immunosuppression tapering experienced fatal outcomes, whereas those with milder symptoms treated with rituximab, antivirals, and/or chemotherapy showed better survival [37].

## 4 HHV‐8/KHSV‐Associated Inflammatory Diseases

### 4.1 Kaposi Sarcoma Herpesvirus Inflammatory Cytokine Syndrome

KICS is a potentially life‐threatening HHV‐8/KSHV‐related inflammatory syndrome characterized by dysregulated host response and cytokine overproduction, particularly interleukin (IL)‐6 and viral IL‐6 (vIL‐6), IL‐1β, IL‐10, tumor necrosis factor (TNF‐α), IL‐17A, and interferon (IFN)‐α [22, 38, 39]. It primarily affects individuals with underlying immunosuppression, such as those with uncontrolled HIV or SOT recipients. Because of its aggressive course and non‐specific clinical presentation, early recognition is essential for timely management, as delayed diagnosis and treatment can rapidly lead to MOF and death.

KICS was categorized by Polizzotto and colleagues in a cohort of 10 HIV‐infected patients with concomitant KS [38]. The diagnosis requires a high plasma HHV‐8/KSHV viral load, elevated C‐reactive protein levels, and the exclusion of MCD in patients with lymphadenopathy. In addition, the patient must exhibit at least two clinical features originating from two or more distinct categories. These categories include symptoms such as fever, fatigue, and respiratory, neurological, or gastrointestinal distress; laboratory abnormalities like cytopenia, hypoalbuminemia, or hyponatremia; and radiographic findings including hepatosplenomegaly, body cavity effusions, or lymphadenopathy [38].

The first case of KICS in a SOT recipient has been described by Mularoni et al. in 2017 [39]. The authors described the case of a liver–kidney transplant recipient with KICS treated successfully with modification of immunosuppression (switch from CNI to mTORi), antivirals and rituximab. The use of anti‐CD20 was already described by Thaunat and colleagues [40] in a case of a donor‐derived HHV‐8/KSHV infection in a kidney transplant recipient who developed, eight months PT, a syndrome characterized by fever, night sweats, weight loss, hepatosplenomegaly, pancytopenia, and positive HHV‐8/KSHV DNA in blood, bone marrow, and pharyngeal samples. The patient improved and survived after administration of four doses rituximab [40].

Earlier reports had already documented cases of inflammatory syndromes, frequently with fatal outcome, associated with HHV‐8/KSHV infection in the SOT population (Table 3). In 2000, Luppi et al. reported the first documented case of donor‐derived HHV‐8/KSHV transmission from a seropositive donor to two seronegative kidney transplant recipients. Four months after transplantation, one recipient developed disseminated KS, which was successfully treated with liposomal doxorubicin, while the other developed a syndrome marked by high fever, splenomegaly, bone marrow failure, and pancytopenia, ultimately dying from MOF despite treatment with antivirals and intravenous immunoglobulins [17]. This case illustrates the intriguing nature of HHV‐8/KSHV infection, which can lead to two distinct diseases, neoplastic in one recipient and inflammatory in the other, in recipients infected with the same viral strain, highlighting the pivotal role of the immune system in the pathogenesis of different KADs.

**TABLE 3:** Reported cases of KICS in SOT recipients.

Number of case, Author, Country, year	Type of Tx (year), serostatus	First DNAemia, time from Tx	Max value DNAemia, time from Tx	Manifestations of HHV‐8/KSHV related Inflammatory Diseases	Exclusion of neoplastic disease	KAD, time from Tx	Therapy	Anti‐CD20 therapy	Attributable death, time from KICS and time from Tx
**KICS ante‐litteram** ^*^									
1. Luppi et al. [17], Italy, 2000	Kidney (1998), D+/R−	50,000– 100,000 cp/mL, 5 mo	50,000– 100,000 cp/mL, 5 mo	Fever, bone marrow failure, anemia, thrombocytopenia, splenomegaly	Yes (bone marrow)	KICS ante‐litteram, 5 mo	Antivirals (ACV, GAN), IVIG	No	Yes, 1 and 6 mo
2. Thaunat et al. [40], France, 2006	Kidney (NS), D+/R−	3000 cp/ 1.5 × 10^4^ PBMCs, 8 mo	7000 cp/ 1.5 × 10^4^ PBMCs, 10 mo	Fever, night sweats, pharyngitis, weakness, weight loss, pancytopenia, hepatosplenomegaly	Yes (bone marrow)	KICS ante‐litteram, 8 mo	IS tapering, steroids (prednisone), RTX	Yes	No
3. Pietrosi et al. [7], Italy, 2011	Liver (NS), D+/R−	380,000 cp/mL, 2 mo	48,000,000 cp/mL, 5 mo	Fever, renal failure, elevated transaminases, ascites, pleural effusion	No	KICS ante‐litteram, 2 mo	Antivirals (CDV)	No	Yes, 4 and 6 mo
4. Pietrosi et al. [7], Italy, 2011	Liver (NS), D+/R−	20,000 cp/mL, 6 mo	2,250,000 cp/mL, 8 mo	Fever, elevated transaminases, renal failure, ascites	Yes (liver, ascites)	KICS ante‐litteram, 6 mo	Antivirals (CDV)	No	Yes, 3 and 9 mo
5. Chiereghin et al. [9], Italy, 2017	Liver (NS), D+/R−	<500 cp/mL, 1.5 mo	98,900 cp/mL, 6 months	Dyspnea, malaise, pancytopenia, pleural and abdominal effusion	Yes (pleural and abdominal effusion)	KICS ante‐litteram, 6 mo	IS discontinuation, antivirals (CDV)	No	Yes, 10 and 11.5 mo
6. Mularoni, et al. [10], Italy, 2025	Liver (2011), D+/R−	106 cp/mL, 1 mo days	231,000 cp/mL, 3.5 mo	Fever, edema, anemia, thrombocytopenia, hypoalbuminemia, splenomegaly, body cavity effusions, elevated CRP	NA	KICS ante‐litteram, 2 mo	Antivirals (CDV)	No	Yes, 5 and 7 mo
7. Mularoni et al. [10], Italy, 2025	Liver (2015), D+/R−	65,000 cp/mL, 3.5	76,000 cp/mL, 4.7 mo	Fever, fatigue, edema, respiratory symptoms, altered mental state, anemia, thrombocytopenia, hypoalbuminemia, hyponatremia, body cavity effusions, elevated CRP	NA	KICS ante‐litteram, 3.5 mo	mTORi, antivirals (FOS)	No	Yes, 1.3 and 4.8 mo
8. Mularoni et al. [10], Italy, 2025	Liver (2016), D+/R−	1475 cp/mL, 2.3 mo	17,000 cp/mL, 2.8 mo	Fever, fatigue, edema, respiratory symptoms, anemia, thrombocytopenia, hyponatremia, body cavity effusions, elevated CRP	NA	KICS ante‐litteram, 3.3 mo	mTORi, antivirals (VGC)	No	Yes, 0.4 and 3.7 mo
**KICS with or without HHV‐8/KSHV‐related neoplastic disease**									
9. Antonio et al. [41], Italy, 2021	Heart (NS), D+/R−	183,673 cp/mL, 11 mo	183,673 cp/mL, 11 mo	Fever, anemia, thrombocytopenia, pleural effusion, lymphadenopathy, elevated CRP	Yes (pleural fluid, bone marrow, lymph node)	KICS and KS of lymph nodes, 11 mo	mTORi, antivirals (VGC, CDV), TOC, CHT (Doxo)	No	No
10. Peri et al. [42], Italy, 2021	Liver (2020), D+/R−	15,490,000 cp/mL, 2 mo	15,490,000 cp/mL, 2 mo	Fever, respiratory failure, anuria, anasarca, coma, elevated interleukin‐6, acute kidney injury, bilateral pneumonia, mediastinal lymphadenopathy, splenomegaly (concomitant SARS‐CoV‐2 interstitial pneumonia)	Yes (bone marrow)	KICS, 2 mo	mTORi, cytokine‐adsorbing hemofilter, RTX	Yes	No
11. Kates et al. [26], U.S., 2024	Liver (NS), suspected D+/R−	4,900,000 million cp/mL, 5 mo	>10,000,000 cp/mL, 6 mo	Fever, malaise, nausea, diarrhoea, weakness, cutaneous violaceous lesions, anemia, pancytopenia, hyponatremia, elevated CRP, pleural effusions, ascites, lymphadenopathies	Yes (pleural and peritoneal fluid, skin)	KICS, disseminated KS and HHV‐8/KSHV related lymphoproliferative disorder, 6 mo	mTORi, antivirals (GAN), RTX	Yes	Yes, 1 and 6 mo
12. Mularoni et al. [10], Italy, 2025	Liver (2018), D−/R−	3469 cp/mL, 4.5 mo	200,000 cp/mL, 6 mo	Fever, fatigue, anemia, thrombocytopenia, hypoalbuminemia, body cavity effusions, elevated CRP	NA	KICS, 6 mo	mTORi, antivirals (CDV, GAN, FOS), RTX	Yes	No
13. Mularoni, et al. [10], Italy, 2025	Liver (2019), D+/R−	4332 cp/mL, 0.7 mo	275,000 cp/mL, 2 mo	Fever, fatigue, edema, respiratory symptoms, anemia, thrombocytopenia, hypoalbuminemia, splenomegaly, body cavity effusions, elevated CRP	NA	KICS, 1.5 mo	mTORi, antivirals (VGC, FOS), steroids, RTX	Yes	No
14. Mularoni, et al. [10], Italy, 2025	Liver (2019), D+/R−	418 cp/mL, 4 mo	52,000 cp/mL, 5.1 mo	Fever, fatigue, respiratory symptoms, anemia, thrombocytopenia, hypoalbuminemia, lymphadenopathy, splenomegaly, body cavity effusions, elevated CRP	Yes	KICS, 4.2 mo	mTORi, antivirals (VGC, FOS), RTX	Yes	No
15. Mularoni et al. [10], Italy, 2025	Liver (2023) D+/R−	535,000 cp/mL, 2.4 mo	535,000 cp/mL, 2.4 mo	Fever, fatigue, edema, anemia, thrombocytopenia, hypoalbuminemia, hyponatremia, elevated CRP	NA	KICS, 2.4 mo	mTORi, antivirals (FOS), RTX	Yes	No
16. Mularoni, et al. [10], Italy, 2025	Lung (2022), D+/R−	1362 cp/mL, 0.6 mo	2,600,000 cp/mL, 13.3 mo	Fever, fatigue, edema, respiratory symptoms, anemia, thrombocytopenia, hypoalbuminemia, hyponatremia, lymphadenopathy, body cavity effusions, elevated CRP	Yes	KICS (11 mo) and disseminated KS (4.4 and fatal relapse at 12 mo)	antivirals (VGC, FOS), CHT (doxo), IVIG, RTX	Yes	Yes, 3 and 14.5 mo
17. Mularoni, et al. [10], Italy, 2025	Liver (2023), D−/R−	418,000 cp/mL, 3 mo	733,000 cp/mL, 3 mo	Fever, fatigue, edema, anemia, thrombocytopenia, hypoalbuminemia, hyponatremia, splenomegaly, body cavity effusions, elevated CRP	NA	KICS, 3 mo	mTORi, antivirals (FOS), Steroids, RTX	Yes	No
18. Knodle et al. [43], USA, 2024	Kidney, (NS)	4400 cp/mL, 14 days	NS	thrombocytopenia, lymphadenopathy, shock	No	KICS and KS	CHT (Doxo), TOC	No	Yes, NS, 34 days
19. Bonazzetti et al. [15], Italy, 2025	Liver,2016, D+/R−	54900 cp/mL, NS	NS	Fever, respiratory symptoms, cachexia, anemia, hypoalbuminemia, serositis, uveitis	Yes	KICS (4 mo)	mTORi, TOC	No	No
20. Bonazzetti et al., Italy, 2025	Heart,2018, D?/R−	183673 cp/mL, NS	NS	Fever, anemia, thrombocytopenia, lymphadenopathy	No	KICS and KS (14 mo)	mTORi, CHT (Doxo)	No	No
21. Bonazzetti et al. [15], Italy, 2025	Liver,2019, NA	114740 cp/mL, NS	NS	Fever, fatigue, anemia, serositys, nephroopaty	No	KICS (13 mo)	mTORi, TOC	No	No
22. Bonazzetti et al. [15], Italy, 2025	Liver, 2021, NA	204850 cp/mL, NS	NS	Fever, respiratory sympthoms, arthralgia, thrombocytopenia, anemia, serositys	No	KICS (10 mo)	No	No	Yes, NS
23. Bonazzetti et al. [15], Italy, 2025	Liver, 2022, D?/R−	>10[10] cp/mL, NS	NS	Fever, diarrhea, anemia, hyponatriemia, serositis	No	KICS and KS (5 mo)	mTORi, TOC	No	No
24. Bonazzetti et al. [15], Italy, 2025	Liver, 2023, D?/R−	>10[10] cp/ml, NS	NS	Fever, respiratory symptoms, edema, anemia, hypoalbuminemia, serositis	No	KICS and MCD (7 mo)	mTORi, CHT (Doxo), RTX, TOC	Yes	Yes, NS
25. Bonazzetti et al. [15], Italy, 2025	Liver−Kidney, 2023, NA	>10[10] cp/mL, NS	NS	Fever, respiratory symptoms, altered mental status, anemia, thrombocytopenia, serositis, lymph nodes	No	KICS (6 mo)	No	No	Yes, NS
**KICS with concomitant or subsequent HLH**									
26. Mularoni et al. [10, 39], Italy, 2017	Liver/ Kidney (2012), D+/R−	7050 cp/mL, 8.6 mo	595,000 cp/mL, 15 mo	Fever, fatigue, edema, anemia, thrombocytopenia, hypoalbuminemia, hypoalbuminemia, hyponatremia, lymphadenopathy, splenomegaly, body cavity effusions, elevated CRP	Yes (lymph nodes)	KICS and HLH, 12.9 mo	mTORi, antivirals (CDV, GAN, FOS), steroids, IVIG, RTX	Yes	No
27. Cona et al. [10, 44], Italy, 2024	Liver (2022), D−/R−	1,000,000 cp/mL, 8.2 mo	7,000,000 cp/mL, 8.8 mo	Fever, fatigue, edema, respiratory symptoms, anemia, thrombocytopenia, hypoalbuminemia, hyponatremia, splenomegaly, body cavity effusions, elevated CRP	NA (H score 256 points)	KICS and HLH (8.2 mo) and subsequent cutaneous and gastrointestinal KS (10.5 mo)	mTORi, antivirals CHT (Doxo), IVIG, steroids RTX	Yes	No

Abbreviations: ACV, Acyclovir; CDV, Cidofovir; CMV, Cytomegalovirus; CHT, Chemotherapy; Cp/ml, copies per milliliters; CRP, C‐reactive protein; D, Donor; Doxo, Doxorubicin; HHV‐8/KSHV, Human Herpes Virus 8/Kaposi's sarcoma‐associated herpesvirus; HLH, hemophagocytic lymphohistiocytosis; KAD, HHV‐8/KSHV‐associated disease; KICS, Kaposi's Sarcoma‐associated Herpesvirus Inflammatory Cytokine Syndrome; KS, Kaposi Sarcoma IS, immunosuppression; IVIG, Intravenous immune globulin; FOS, Foscarnet; GAN, Ganciclovir; mo, Months; mTORi, mammalian target of rapamycin inhibitors; NA, Not applicable; NS, Not specified; PBMCs, Peripheral blood mononuclear cell R, Recipient; RTX, Rituximab; TOC, Tocilizumab; Tx, Transplant; VGC, Valganciclovir.

* KICS ante‐litteram was defined in consideration of the date of the event.

Chiereghin et al., documented one fatal case of MOF in a SOT recipient with a non‐malignant HHV‐8/KSHV‐related illness, preceded by fever, pancytopenia, and effusions [9]. In 2011 Pietrosi et al., among 8 SOT recipients with D+/R‐ mismatch, described 4 donor‐derived infections, 2 of whom experienced a lethal non‐malignant illness, following treatment with only antivirals [7].

Nowadays, all these cases could be classified by using the term of “*KICS ante‐litteram*” as defined by syndromes that fulfill the KICS criteria of Polizzotto et al. but described before 2016.

After 2016, two additional cases of KICS in SOT recipients successfully treated by controlling the cytokine storm, using either rituximab or tocilizumab, or a cytokine‐adsorbing hemofilter have been described, suggesting that prompt recognition and treatment addressing the immune dysregulation may be associated with better outcome [41, 42].

Between 2015 and 2021, several cases of fatal primary HHV‐8/KSHV infection in SOT recipients in UK have been reported, including a cluster of 27 recipients. Transmission and mortality rates were 52% and 57%, respectively [45]. Following analysis of these cases, a recommendation for universal screening of deceased donors was made, sending samples to a reference laboratory for testing [45].

In a recently published study conducted by our group, including 1856 SOT recipients and 1349 donors, seroprevalence was 8.4% among recipients and 3.3% among donors. Among 49 patients with mismatch (D+/R−), the rate of HHV‐8/KSHV transmission was 45%. During the study period, a total of 11 KICS were observed as result of a primary infection, (including 1 case of KICS with disseminated KS and another case of KICS, followed by hemophagocytic lymphohistiocytosis [HLH] and disseminated KS) [10, 44]. Before 2017, when management relied primarily on antivirals, mortality in KICS was 75%. After implementing our risk mitigation protocol, consisting of switch from CNI to mTORi and foscarnet in case of persistent positive DNAemia, and prompt administration of rituximab in case of KICS, mortality was 14%: Only one patient, for which switch to mTORi was deemed as not feasible, died for concomitant disseminated KS and KICS. Despite the non‐randomized study design and the small sample size, this was the first study to suggest that a risk mitigation protocol and the systematic use of rituximab for KICS is associated with improved outcomes in SOT recipients [10].

Recently Bonazzetti and colleagues performed a systematic review of the literature: The overall mortality among KICS patients was 35% (5 out of 14). Notably, mortality was substantially lower (1/10, 10%) among those treated with rituximab (*n* = 9) or tocilizumab (*n* = 1) compared with untreated patients (4/4, 100%), suggesting the therapeutic benefit of immunomodulatory therapy in this setting [15].

**FIGURE 2:**
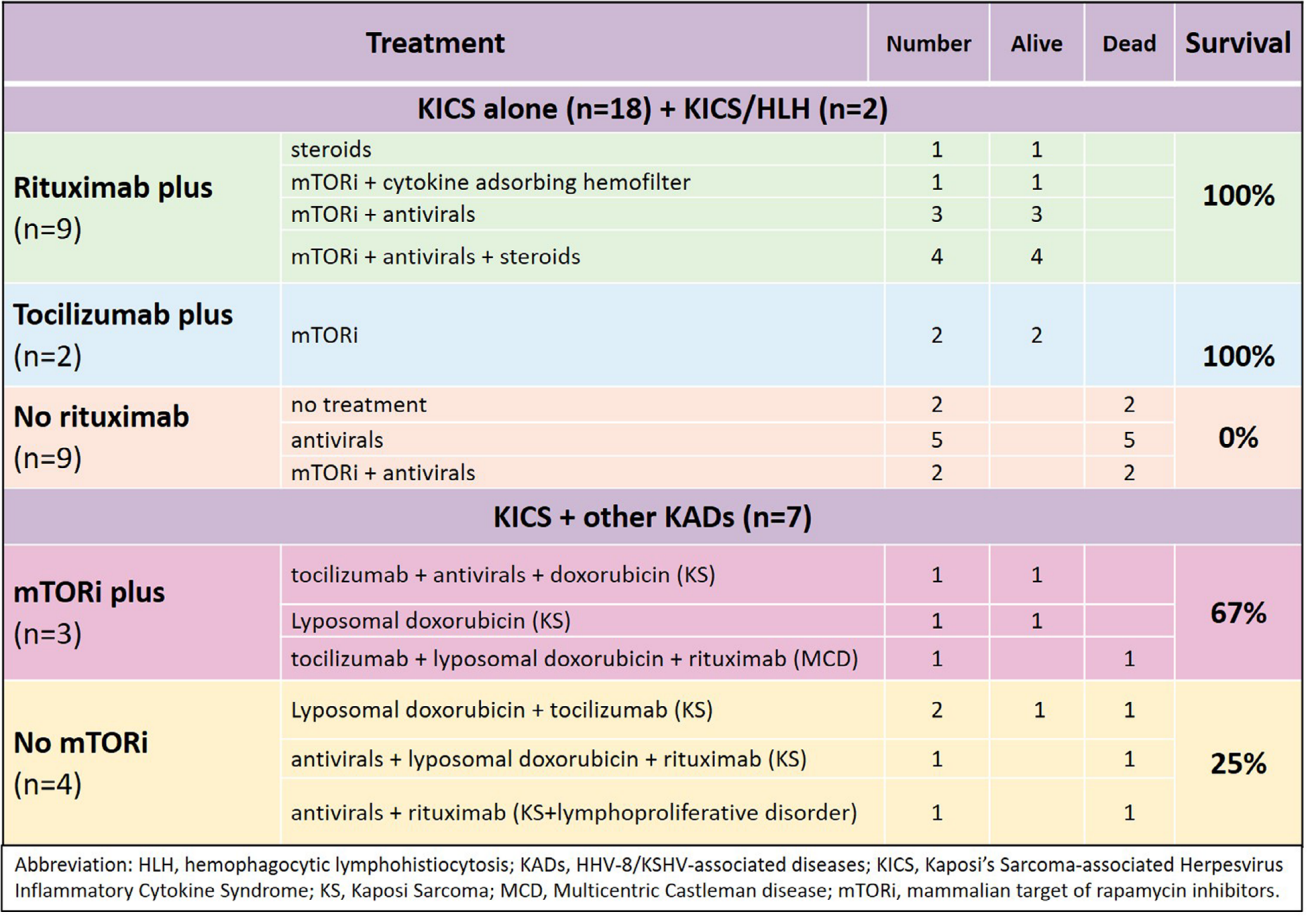
Treatment of all reported cases of KICS and survival rate.

In our literature search, we found a total of 27 cases of KICS in SOT recipients, 8 of them occurring before 2016, year of the working case definition by Polizzotto et al., and therefore categorized as “KICS ante‐litteram”. Of them, 18 recipients had KICS alone and 2 had KICS plus HLH: Rituximab therapy was associated with 100% survival (7/7) as for tocilizumab 100% (2/2) compared to 0% when neither rituximab nor tocilizumab were used (Figure 2).

Seven KICS occurred with concomitant KADs, switch to mTORi appears to be associated with better outcome, however numbers are small and timing in initiating an appropriate therapy could be an important factor influencing the outcome.

The antiviral effects of mTORi have been linked to improvement in T‐cell functionality/memory, by regulating the RTA expression that triggers lytic replication [46]. Pharmacological inhibition of mTOR exerts strong anti‐neoplastic activity in cells or tumors dependent on elevated mTOR activity as in HHV8/KSHV‐infected cells [46]. Antivirals can be used, however data supporting their efficacy are lacking. The most promising treatment for KICS is the use of rituximab: Its use is rationally grounded in the targeted elimination of proliferating HHV‐8/KSHV–infected mature B cells (CD20^+^), thereby reducing the viral burden and cytokine levels. A delay in initiating rituximab therapy, in this clinical setting, may lead to fatal outcomes, which cannot be prevented by the use of antiviral agents alone.

### 4.2 KICS and Hemophagocytic Lymphohistiocytosis

HLH, similarly to KICS, is characterized by an unregulated and exuberant systemic immune response and can be either primary, on a genetic basis, or secondary due to malignancies, autoimmune disorders, iatrogenic factors, or triggered by viral infections. In a recent retrospective cohort study by Miguad et al. comprising 22 HIV‐infected patients with HLH, HHV‐8/KSHV was the trigger in 11 out of 22 cases (50%) [47].

During KICS, a concomitant HLH should be suspected in the case of worsening of patient's clinical conditions, bone marrow failure and organ damage, with or without increase in HHV‐8/KSHV viral load, despite appropriate KICS therapy [44].

Diagnosis of HLH, as recommended by the HLH steering committee, requires the presence of at least five out of eight diagnostic criteria which includes fever, cytopenia, hepatomegaly or splenomegaly, hemophagocytosis signs in bone marrow, lymph nodes or spleen, hyperferritinemia, hypofibrinogenemia, hypertriglyceridemia, low or absent NK‐cell activity, and increased soluble IL‐2 receptor [48, 49] (Table 4).

**TABLE 4:** Differential diagnosis and treatment between KICS and HHV‐8/KSHV‐associated hemophagocytic lymphohistiocytosis.

KICS	HLH
**Diagnosis**	
**Diagnosis of KICS established if present at least 2 clinical manifestations from at least 2 categories (1a, 1b, and 1c), together with each of the criteria in 2, 3, and 4**	**Diagnosis of HLH established if Criterion 1 or 2 is fulfilled (5 of the 8 criteria)**
Clinical manifestations: Symptoms (Fever, fatigue, edema, cachexia, respiratory symptoms, gastrointestinal disturbance, neuropathy) Laboratory abnormalities (anemia, thrombocytopenia, hypoalbuminemia, hyponatremia) Radiographic abnormalities (lymphadenopathy, splenomegaly, hepatomegaly)	1. A molecular diagnosis consistent with HLH
2. Evidence of systemic inflammation (elevated C‐reactive protein ≥3 g/dL)	2. Diagnostic criteria for HLH: − fever − splenomegaly − cytopenias (affecting >=2 of 3 lineages in the peripheral blood) Hemoglobin, 90 g/LPlatelets <100 x 10^9^/LNeutrophils <1.0 x 10^9^/L − hypertriglyceridemia and/or hypofibrinogenemia − hemophagocytosis in bone marrow or spleen or lymph nodes. No evidence of malignancy − low or no NK cell activity − ferritin >= 500 mg/L − sCD25 >=2400 U/mL
3. Elevated KSHV viral load in plasma (≥1000 copies/mL) or peripheral blood mononuclear cells (≥100 copies/10^6^ cells)	
4. No evidence of KSHV‐associated multicentric Castleman disease	
**Treatment**	
IS reduction, Switch to mTOR	Dexamethasone +/− etoposide
Rituximab	Treatment of the triggering condition
+/− Antivirals	+/− intravenous immune globulin or inhibitors of interleukin‐1

The distinction of KICS and HLH has important therapeutic and management implications. Differently from KICS, treatment algorithm for HHV‐8/KSHV associated HLH may include high dose of dexamethasone, and eventually etoposide; the treatment of the triggering condition should be provided. In addition, intravenous immune globulin, inhibitors of interleukin‐1, interferon‐γ, and Janus kinases can be considered (Table 4) [24, 50].

## 5 Cytokine Expression Patterns and Specific T‐Cell Response During KADs

In SOT recipients, immune profiling across the spectrum of KADs, from asymptomatic DNAemia to KS and KICS reveals a dynamic continuum of immune activation and exhaustion [10]. In asymptomatic cases, low‐level DNAemia is accompanied by modest cytokine release. This controlled state prevents disease manifestation, despite viral latency, indicating that HHV‐8/KSHV reactivation alone is insufficient to drive pathology without concomitant immune dysregulation [51]. By contrast, KICS represents a state of dysregulated immune activation, characterized by a systemic “cytokine storm” dominated by IL‐6, IL‐10, TNFα, IFNα, and soluble CD14. Despite this heightened inflammatory response, KICS patients often exhibit early signs of immune exhaustion, including increased expression of inhibitory receptors such as PD‐1 and LAG‐3 [51].

Patients with KS display profound immune exhaustion and loss of HHV‐8/KSHV‐specific T cell function. The associated cytokine milieu, rich in IL‐6, IFNα, CD163, and hepatocyte growth factor (HGF), defines a tumor‐promoting and immunosuppressive microenvironment that supports angiogenesis, macrophage M2 polarization, and endothelial proliferation. This shift from inflammation to oncogenesis highlights the interplay between chronic immune activation, tissue remodeling, and impaired antiviral defense [51].

Functional analyses consistently demonstrate that HHV‐8/KSHV‐specific T cell responses are more robust in asymptomatic individuals and progressively weaken in KICS and KS, mirroring clinical severity [51]. Longitudinal monitoring of interferon‐γ‐producing T cells shows that sustain antiviral responses correlate with viral control and favorable outcomes. The upregulation of PD‐1, LAG‐3, and VISTA confirms immune exhaustion, as a central mechanism driving viral persistence, suggesting that checkpoint inhibition could help restoring antiviral activity.

Therapeutically, these insights point toward distinct yet complementary strategies. In KICS, targeting CD20 B cell depletion with rituximab, and eventually block IL‐6–driven inflammation through cytokine inhibitors, may help mitigate hyperinflammation and rebalance immune activation. Immune checkpoint blockade directed against PD‐1 or LAG‐3 represents a promising approach to reverse T cell dysfunctions. In KS, therapeutic efforts should focus on remodeling the tumor microenvironment, through inhibition of the HGF/c‐Met axis or depletion of CD163‐positive tumor‐associated macrophages, to curb angiogenesis and tumor progression. Importantly, because clinical deterioration in HHV‐8/KSHV‐related diseases often parallels a profound impairment of T cell function, the adoptive transfer of virus‐specific cytotoxic T lymphocytes (CTLs) could be explored as a promising therapeutic strategy. Virus‐specific CTLs have already demonstrated efficacy against other herpesvirus infections in SOT recipients and could restore targeted antiviral immunity, and prevent progression to KICS or KS [52]. Finally, combining cytokine profiling, checkpoint expression analysis, and longitudinal T cell monitoring may allow for more precise risk stratification and tailored immunotherapy in this high‐risk population.

## 6 Current Gaps of Knowledge of HHV‐8/KSHV in SOT

Despite growing recognition of HHV‐8/KSHV as an important pathogen in SOT recipients, substantial gaps persist.

### 6.1 Lack of Standardized Screening and Management Protocols

In the absence of standardized screening and monitoring protocols for HHV‐8/KSHV in SOT, current guidelines do not recommend universal pre‐transplant screening of donors or recipients. Nonetheless, emerging evidence indicates that risk stratification, increased awareness, prompt recognition, and early therapeutic intervention can significantly mitigate the clinical impact of HHV‐8/KSHV infection [10, 16, 26]. Similarly, therapeutic approaches for KICS, such as switch to mTORi, antiviral treatment, and rituximab administration should be studied in prospective studies. However, these will be challenging given the rarity of HHV‐8/KSHV‐related disease in SOT [10, 25, 40].

### 6.2 Poorly Defined Natural History and Clinical Manifestations in SOT Recipients

While KADs as KS, PEL, and MCD are well‐characterized in the HIV population, in which the majority of studies were conducted, the natural history of HHV‐8/KSHV infection and related complications in SOT remains poorly understood.

The prevalence and distribution of KADs in the SOT setting may differ from the one observed in other immunocompromised populations such as HIV patients or HSCT recipients. In fact, KS is more common in HIV‐infected individuals than in SOT recipients and other neoplastic manifestations have been rarely reported in SOT [21]. KICS is increasingly reported SOT recipients with mismatch (D+/R−) mainly following donor‐derived primary infection [10].

Some patients experience overlapping or sequential KADs (e.g., KS followed by MCD or PEL, or KICS with HLH), especially in HIV infected individuals [53]. Finally, there is a paucity of data on predictive factors, progression timelines, or long‐term outcomes in the SOT context.

### 6.3 Generalizability of KICS Criteria to the SOT Population

KICS was first described by Polizzotto et al. in 10 HIV‐positive individuals with concomitant KS, high HHV‐8/KSHV viremia, and systemic inflammation [38]. Therefore, the applicability of this classification to SOT recipients remains uncertain. Indeed, immunosuppressive therapy in this population may blunt inflammatory responses, obscure clinical signs, or produce laboratory abnormalities that mimic other conditions such as rejection or infection.

Further studies are therefore needed to better define the clinical, laboratory, and radiologic characteristics of KICS in the SOT population and to develop transplant‐specific diagnostic criteria.

## 7 Risk Mitigation Strategies

**FIGURE 3:**
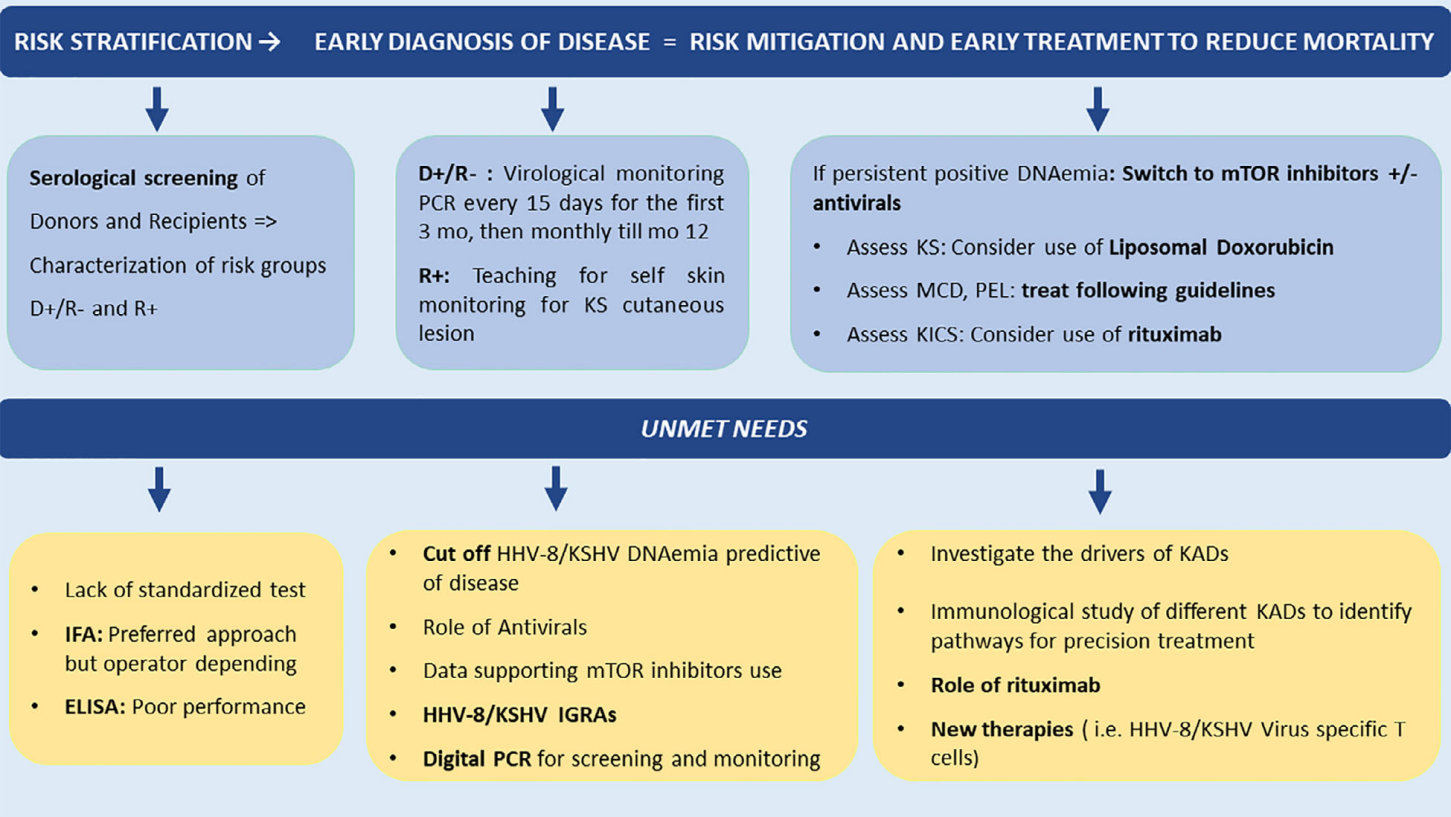
Mitigation strategy and unmet needs.

1. Risk assessment based on serological match of donors and recipients is the key to identify the highest‐risk group for close clinical and virological surveillance and to implement risk mitigation strategies (Figure 3). We believe that screening of donor and candidate for HHV‐8/KSHV is crucial for optimizing PT outcomes [54]. However, lack of an optimal serological assay is the major limitation to implement this recommendation. Two main assays are used for HHV‐8/KSHV serological screening: Immunofluorescence assay (IFA) and enzyme‐linked immunosorbent assay (ELISA) [9]. Single‐antigen ELISA tests have poor sensitivity and specificity, while IFA using lytic and latent antigens perform better but are labor‐intensive and require expert interpretation. However, data on SOT populations are scarce and sometimes conflicting (Tables 1 and 2).
2. Clinical and virological monitoring of at‐risk recipients. Similar to EBV, in D+/R− the appearance of DNAemia usually precedes clinical manifestation and during this time window the clinician could have the opportunity to put in place interventions to reduce the impact of infection and mitigate disease severity (Figure 3).
3. In D+/R− when DNAemia is persistently positive, suggesting that patient's immune system is unable to control the infection, the immunosuppression should be reduced and the regimen modified by replacing CNIs with mTORi, when feasible. mTORi regulate RTA expression and, therefore, HHV‐8/KSHV lytic replication, have direct anti‐proliferative and anti‐angiogenic effect and promote recovery of specific HHV‐8/KSHV T cell response [29, 55]. Some clinicians report the use of antiviral therapy with foscarnet, cidofovir, or (val)ganciclovir, however there is a paucity of data to support this recommendation.
4. In patients with KICS, rituximab should be administered as soon as the condition is diagnosed (Figure 3). Rituximab depletes HHV‐8/KSHV‐infected B cells, thereby reducing cytokine levels. High clinical suspicion in recipients at risk based on serological status can elicit prompt suspicion of MCD, PEL, and other KADs in case of suggestive symptoms. In certain cases, multiple KADs may occur simultaneously in SOT recipients. Notably, rituximab has been linked to flare‐ups of KS in HIV‐positive patients [56], therefore clinicians must be aware of this risk. Caution is essential when KICS and KS coexist and, if both conditions are present, liposomal doxorubicin and rituximab should be administered together [24].
5. In HHV‐8/KSHV Ab positive recipients, besides clinical and virological monitoring, teaching for self‐detection of Kaposi‐like skin lesions may be provided along with dermatological follow‐up (Figure 3). In case of suspected lesions, if cutaneous KS is confirmed, staging with endoscopy/imaging should be performed, switch to mTORi should be applied, and liposomal doxorubicin is introduced if visceral KS is diagnosed.

## 8 To Screen or Not to Screen? Hamletic Question! (Pros and Cons)

CONS: *The incidence of* PT *KS and other KADs is low, which is then the usefulness of a screening protocol, if most patients at high or intermediate risk, develop no clinical manifestations of* KAD.
PROS: KICS is a complex and rapidly fatal condition which could be underdiagnosed and mistaken for other causes of sepsis, with negative blood cultures; thus, increased awareness of the risks based on pre‐transplant screening might help to increase accurate and timely diagnosis and ensure better outcomes.
CONS: *Seroprevalence is low in most countries, and screening of donors and recipients may not be judged as cost‐effective in areas with a lower seropositivity rate*.
PROS: As the characteristics of organ donors evolve, including increasing numbers of transplants performed from donors with HIV infection, MSM, or history of injection or inhalation drugs use, HHV‐8/KSHV seroprevalence among donors is likely to exceed national averages, in non‐endemic geographic areas.
CONS: *Even in settings with higher‐than‐expected prevalence, the absence of standardized treatment protocols for management of KADs limits the clinical utility of identifying seropositive individuals. So, which is the usefulness of a screening if it cannot mitigate the disease?*
PROS: Based on recent data, early diagnosis, and prompt treatment with immunosuppressive modification (switch to mTORi) and rituximab has improved the outcome of donor‐derived HHV‐8/KSHV primary infections and KICS in SOT.
CONS: *HHV‐8/KSHV antibody testing itself is technically challenging. The assays are complex and limited in availability. Screening donors before transplantation may not be feasible, as organ procurement organizations (OPOs) typically lack access to timely HHV‐8/KSHV testing before organ allocation, further reducing the practicality of implementing widespread screening protocols*.
PROS: Since onset timing of donor‐derived HHV‐8/KSHV primary infections is usually within the first 2–4 months following transplantation, PT donor testing is a feasible approach, with the option to send samples to reference laboratories and obtain serology results within the first month after SOT. A reliable HHV‐8/KSHV serology should be available for clinical research and for identifying high‐risk patients. With increased awareness and clinical need, assay development and availability may change in the future, allowing expansion of testing strategies.

Based on the latest evidence, severe cases of donor‐derived HHV‐8/KSHV infection, often lacking cutaneous manifestations and sometimes identified only at autopsy, underscore the need to enhance clinicians’ awareness of this condition. PT monitoring of patients at high risk (D+/R−) or intermediate risk (R+) for KADs allow for early recognition and prompt management, which are crucial for achieving better outcomes in SOT.

However, the absence of standardized serologic assays remains the primary limitation to widespread implementation of such screening strategies. To validate these findings and recommendations, multicenter prospective studies are urgently needed.

## 9 Future Directions

Despite recent advances in the understanding and management of HHV‐8/KSHV infection in SOT recipients, several key questions remain unanswered (Figure 3).

First, a standardized serological test for screening should be made available, and a viral load cut‐off predictive of disease should be established. The role of digital PCR should be explored both for screening and for monitoring protocols and harmonize management strategies across transplant centers.

Second, the mechanisms driving HHV‐8/KSHV infection toward neoplastic or inflammatory disease remain unexplored. Comparative immunological profiling could help identify key pathways and guide interventions. An HHV‐8/KSHV Interferon Gamma Released Assay (IGRA) could be a useful tool to guide clinicians to modulate immunosuppression and predict the evolution to KS as an inverse relationship between detection of virus‐specific T‐cell responses and virus control has been reported for almost all herpes viruses‐associated diseases.

Third, the use of mTORi represents a rational therapeutic strategy. However, immunological and T cell response data would define their optimal timing, dosing, and combination with antiviral therapy, in both prophylactic and therapeutic settings.

Furthermore, the potential role of newer antivirals should be explored, for instance Maribavir by inhibiting transcription of EBV, a gamma‐herpesviruses like HHV‐8/KSHV could theoretically exert similar antiviral effects against HHV‐8/KSHV.

Rituximab, when administered in the early phase, has shown efficacy in KICS. However, further studies are needed to confirm the efficacy of selective cytokine blockade by tocilizumab, in the case of poor response to rituximab in patients with KICS. Moreover, the reasons behind the failure in CTLs expansion are unclear. Caduff et al., has recently reported that KSHV/EBV co‐infection of mice with reconstituted human immune systems (humanized mice) leads to IgM responses against both latent and lytic HHV‐8/KSHV antigens, and expansion of central and effector memory CD4+ and CD8+ T cells. Among these, KSHV/EBV dual infection allows for the priming of CD8+ T cells that are specific for the lytic HHV‐8/KSHV antigen K6 and able to kill KSHV/EBV infected B cells, suggesting that K6 may be either a potential vaccine antigen or a good target for CTLs therapy [57].

Altogether, by addressing these gaps through prospective, interventional multicentre studies will be crucial to establish standardized diagnostics, unravel the immunopathogenesis of HHV‐8/KSHV‐related diseases, and develop tailored therapeutic strategies to reduce morbidity and mortality in SOT recipients.

## Funding

This work was funded by the Italian Ministry of Health (Ricerca Corrente 2025, Linea 2), and by EU funding within the NextGenerationEU—MUR—National Recovery and Resilience Plan, Mission 4, Component 2 Investment 1.3 – Extended Partnership initiative on Emerging Infectious Diseases INF‐ACT (project no. PE00000007, CUP B73C22001230006) to A.M. and CN3 Terapia Genica‐Spoke 2 (project no. CN00000041) to M.L.

## Consent

The authors have nothing to report.

## Conflicts of Interest

The authors declare no conflicts of interest.

## Data Availability

Data sharing not applicable to this article as no datasets were generated or analyzed during the current study.
